# Liver Abscess Caused by Ingestion of a Sewing Needle

**DOI:** 10.7759/cureus.8924

**Published:** 2020-06-30

**Authors:** Ryan L Basquez, Ifrah Butt, Ashley Billings, Micah Pippin

**Affiliations:** 1 Family Medicine, Rapides Regional Medical Center, Alexandria, USA; 2 Internal Medicine, Graduate Medical Education, Aventura Hospital, Aventura, USA

**Keywords:** foreign bodies, liver abscess

## Abstract

Liver abscesses have a low incidence and can be caused by multiple etiologies. We present an interesting case of a 74-year-old woman who presented with abdominal pain and leukocytosis. Imaging revealed a foreign body in the antrum of the stomach extending to an area of hypodensity in the liver. She was taken for an exploratory laparotomy and was found to have a gastric perforation due to a sewing needle. The needle had embedded in the liver causing the formation of an abscess. The patient was successfully treated with surgical management and a course of antibiotics.

## Introduction

Liver abscesses have a relatively low incidence, estimated at 2.3/100,000 per year, and can be caused by multiple etiologies including complications of surgery (such as those of the biliary tract and liver), penetrating trauma, foreign bodies, parasites, and more [1,2]. Presenting signs and symptoms include right upper quadrant (RUQ) pain and tenderness, fever, chills, rigors, jaundice, icterus, hepatomegaly, splenomegaly, and cough [3]. Treatment options vary depending on the underlying etiology, as well as the size and number of abscesses present. Options for drainage include percutaneous ultrasound- or CT-guided aspiration, endoscopic retrograde cholangiopancreatography (ERCP), or surgically either laparoscopically or open.

## Case presentation

**Figure 1:**
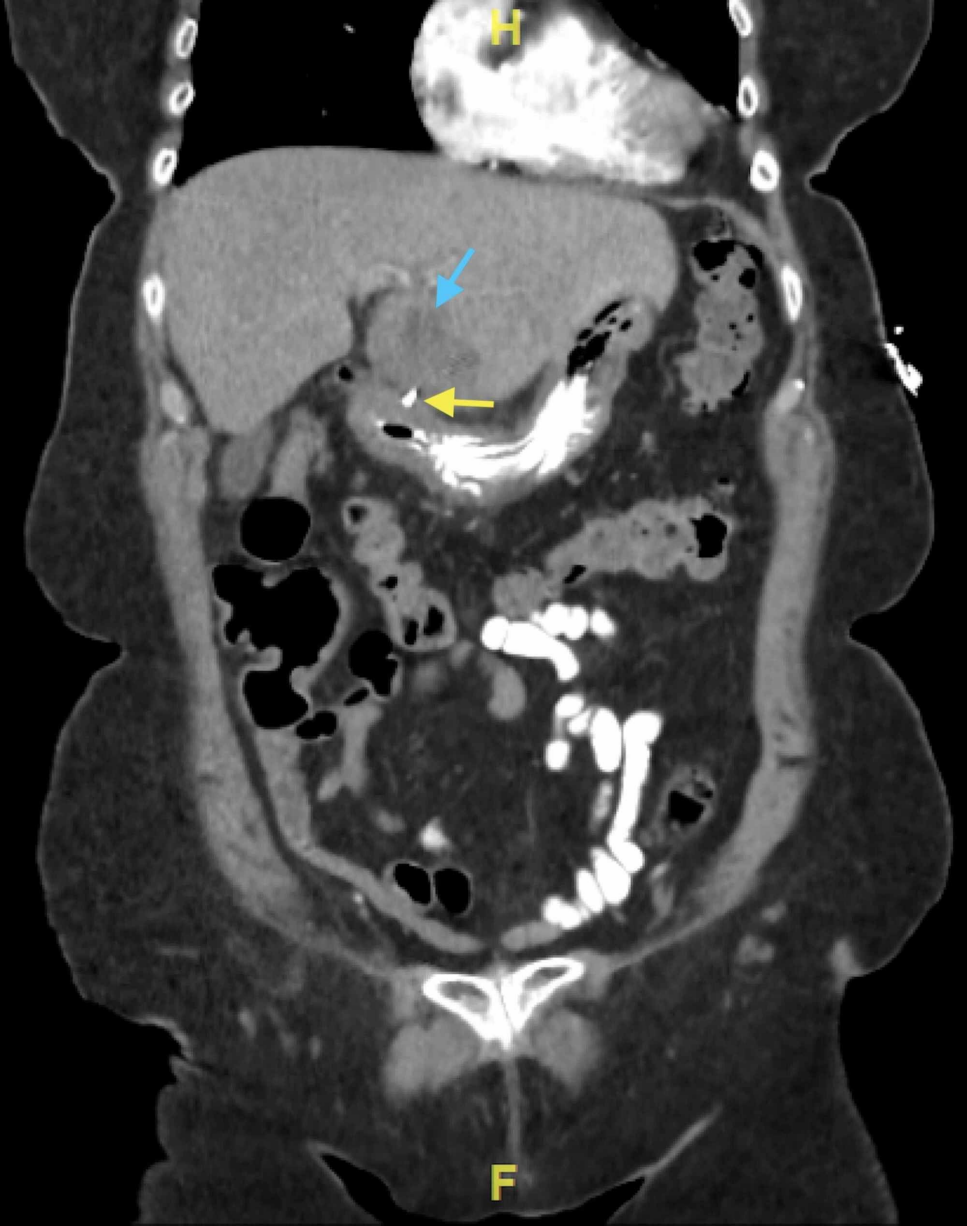
A coronal view of CT abdomen and pelvis showing a metallic foreign body (yellow arrow) penetrating the gastric antrum into a hypodense structure in the liver (blue arrow)

**Figure 2:**
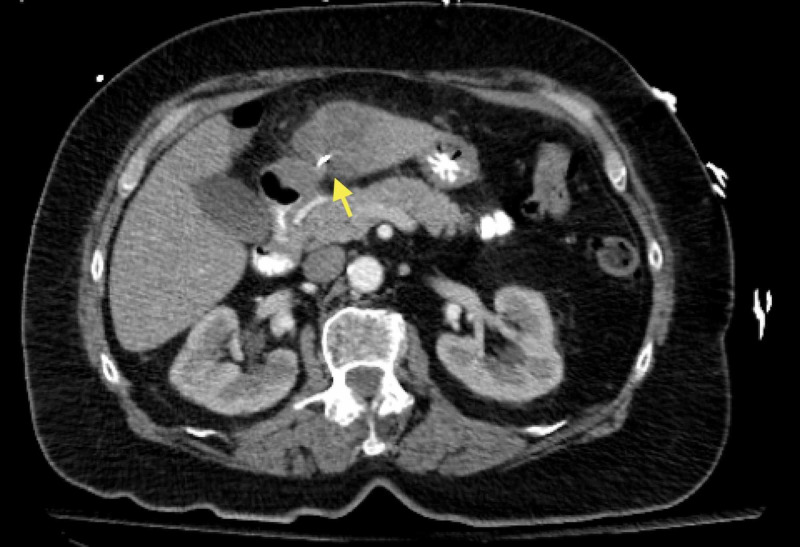
An axial view of the CT abdomen and pelvis showing the metallic foreign body penetrating the liver (yellow arrow)

**Figure 3:**
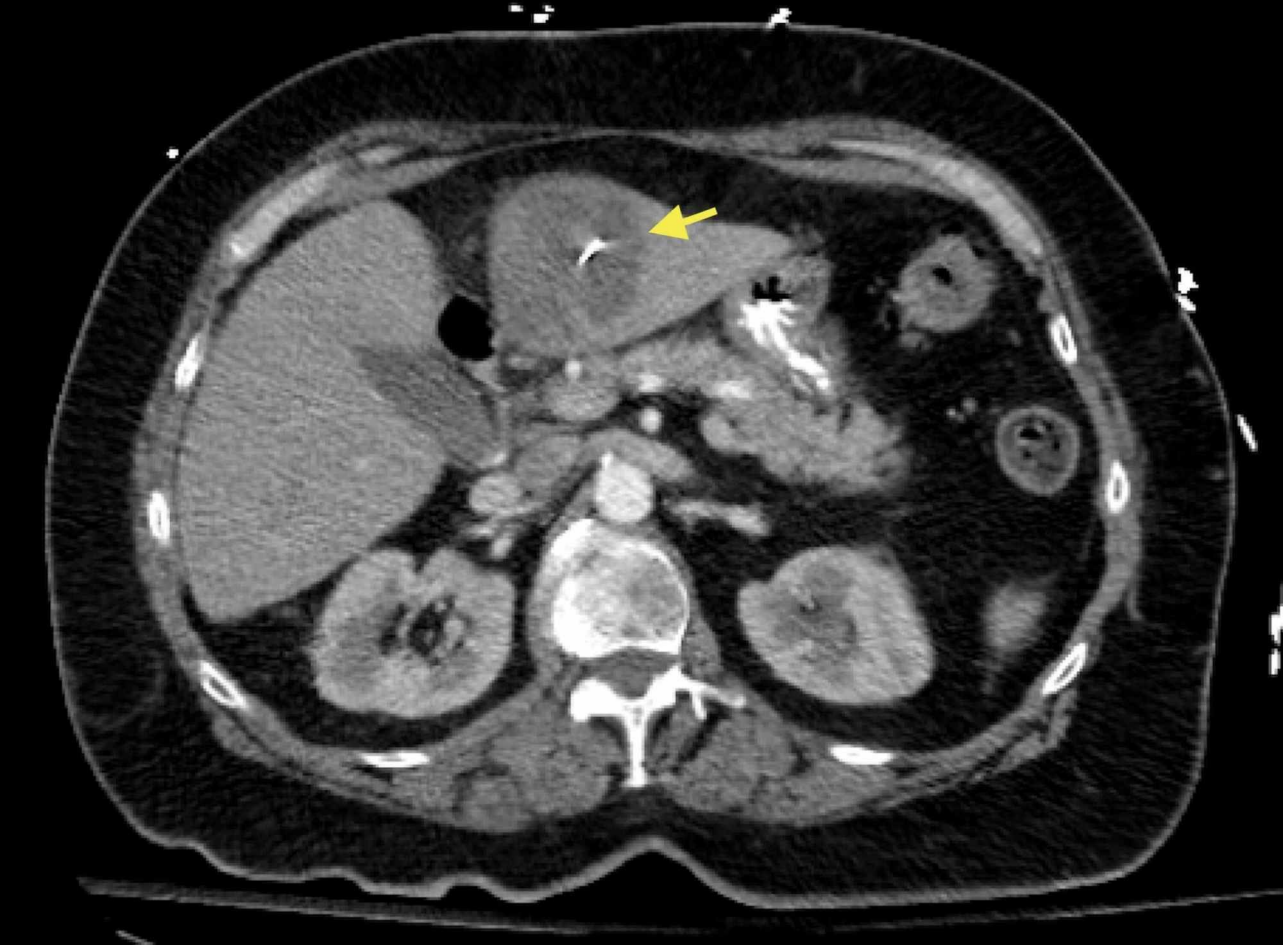
Another axial view, proximal to the above image (Figure 2), showing the metallic body within the hypodense area of the liver (yellow arrow)

A 74-year-old Caucasian woman with hypertension, hyperlipidemia, diabetes mellitus type 2, diverticulosis, and a prior appendectomy presented to the ED with several weeks of moderate to severe, intermittent, RUQ and epigastric abdominal pain. She described the pain as a "stabbing" sensation, non-radiating, and acutely worsening in severity and frequency over the past week. Her physical examination was significant for epigastric and RUQ tenderness on palpation without rigidity, rebound, or guarding. She denied fever, chills, myalgias, nausea, and vomiting. Her temperature was 97.8°F and she remained afebrile throughout her hospital stay. Her initial laboratory data is depicted in Table 1. Ultrasound revealed an ill-defined hypodense area in the anterior inferior left hepatic lobe. A CT scan of the abdomen and pelvis with intravenous and oral contrast showed a large, hypodense mass in the left lobe of the liver with a faint enhancement measuring 4.7 x 4.5 cm and a metallic foreign body extending from the distal antrum of the stomach wall into the hypodense area within the liver (Figures 1-3). 

**Table 1:** Patient's laboratory data on initial presentation

Laboratory Data	
White blood cells (WBC)	17.9 k/mm^3
Neutrophils	75.60%
Total Bilirubin	1.5 mg/dL
Aspartate transaminase (AST)	37 Units/L
Alanine transaminase (ALT)	29 Units/L
Alkaline phosphatase	103 Units/L

The patient was started on broad-spectrum empiric antibiotics and was taken for urgent surgery. She underwent an exploratory laparotomy, removal of the foreign body, which was found to be a sewing needle, surgical drainage of the abscess, and repair of the gastric perforation. Streptococcus viridans was later isolated in the fluid cultures obtained during surgical drainage. Post-operatively, her abdominal pain had significantly improved and her leukocytosis resolved. She tolerated the procedure well, clinically improved, and was discharged home with a 14-day course of antibiotics.

## Discussion

Pyogenic liver abscesses are a relatively uncommon condition with a significant mortality rate of 2%-12% [4]. Seeding of pathogens can occur via the bile ducts, blood vessels (arterial or portal system), or by contiguous spread from an area of infection [5]. The etiologies of liver abscesses are shown in Table 2 [5]. Common pathogens include Streptococcus spp., gram-negative organisms like Escherichia coli and Klebsiella pneumoniae, and anaerobic organisms. Other pathogens, including Candida spp., Pseudomonas aeruginosa, and Staphylococcus aureus are uncommon and usually detected due to specific etiologies or in a particular context [4]. For example, Staphylococcus aureus is more commonly due to penetrating trauma or chemoembolization [6].

**Table 2:** Etiologies of pyogenic liver abscesses

Etiologies of Pyogenic Liver Abscesses	
Biliary Disease	cholangitis
	cholecystitis
	obstructing tumors (e.g., cholangiocarcinoma)
	biliary strictures
Bowel infections	appendicitis
	diverticulitis
Underlying inflammatory bowel disease	Crohn's disease
Iatrogenic causes	post-liver transplant
	biliary stent complications
	interventional techniques (e.g. intra-arterial chemoembolization, radiofrequency ablation)
Direct extension from another site of infection	subphrenic abscess
	perinephric abscess
Bacteremia	
Ischemia	
Penetrating Liver trauma	

The clinical presentation of pyogenic liver abscesses is non-specific; hence, a high index of suspicion is necessary for prompt diagnosis [7]. In a 10-year case review conducted by Rahimian et al., the most common symptoms included fever, chills, and RUQ pain or tenderness [8]. Other symptoms can include rigors, nausea, vomiting, weight loss, malaise, and anorexia [7]. The most common laboratory abnormalities are an elevated WBC, temperature > 100.4°F, a low albumin level, and an elevated alkaline phosphatase level (in 67%-90% of patients) [7,8]. Approximately 50%-65% of patients can have elevated AST, ALT, and total bilirubin levels [7,9,10]. Patients can also have positive blood cultures; however, this is not always the case. In a study conducted by Barnes et al., which examined features of pyogenic abscesses in 48 cases, abscess cultures yielded pathogens in 90% of the cases, and blood cultures were positive in 50% of the cases [11]. Elevated inflammatory markers, including erythrocyte sedimentation rate (ESR) and c-reactive protein (CRP), may also be seen and are sensitive but non-specific [7].

CT or ultrasound is the preferred imaging modalities to diagnose hepatic abscesses. Usually, they appear as non-enhancing, hypodense lesions with an enhancing ring on CT and as hypo- or hyperechoic lesions with internal debris on ultrasound [7]. 

Treatment involves abscess drainage, foreign body extraction (if present), and targeted antibiotic therapy. With the evolution and advances in interventional radiology, the preferred method of drainage is via the percutaneous route. Criteria for percutaneous drainage include abscess size >5 cm, persistent fevers despite 48-72 hours of appropriate medical therapy, or concerning clinical or imaging features that may suggest impending perforation [7]. Surgery should be considered in patients that have a large or multiloculated abscess, had an inadequate response to therapy after percutaneous drainage, or if there is abscess rupture [10]. Other options for abscess drainage include needle aspiration via ERCP if the biliary origin is suspected. Our patient was successfully treated with surgical abscess drainage as she had a penetrating foreign body that caused a gastric perforation, in addition to the abscess formation.

## Conclusions

To conclude, clinicians must have a high index of suspicion to diagnose and treat hepatic abscesses effectively. RUQ pain, fever, leukocytosis with or without abnormal liver enzymes should alert clinicians to the possibility of a liver abscess. Abdominal ultrasound or CT imaging is the preferred modalities for diagnosis. Percutaneous drainage, in conjunction with targeted antibiotic therapy, is the mainstay of treatment. Although outcomes have improved over the years, delayed diagnosis or treatment can be detrimental to the patient as this condition is known to have high mortality rates if inadequately treated.

## References

[1] Akhondi H, Sabih DE (2020). Liver Abscess. Updated.

[2] Mohsen AH, Green ST, Read RC, McKendrick MW (2002). Liver abscess in adults: ten years experience in a UK centre. QJM.

[3] Gupta SK, Rasool A, Hela AH, Goel R, Hussain Z (2019). Clinical profile and management of pyogenic liver abscesses in a tertiary care hospital. Int J Res Med Sci.

[4] Mischnik A, Kern WV, Thimme R (2017). Pyogenic liver abscess: changes of organisms and consequences for diagnosis and therapy. Dtsch Med Wochenschr.

[5] Lardière-Deguelte S, Ragot E, Amroun K (2015). Hepatic abscess: diagnosis and management. J Visc Surg.

[6] Chen C, Chen PJ, Yang PM (1997). Clinical and microbiological features of liver abscess after transarterial embolization for hepatocellular carcinoma. Am J Gastroenterol.

[7] Longworth S, Han J (2015). Pyogenic liver abscess. Clin Liver Dis.

[8] Rahimian J, Wilson T, Oram V, Holzman RS (2004). Pyogenic liver abscess: recent trends in etiology and mortality. Clin Infect Dis.

[9] Zhu X, Wang S, Jacob R, Fan Z, Zhang F, Ji G (2011). A 10-year retrospective analysis of clinical profiles, laboratory characteristics and management of pyogenic liver abscesses in a Chinese hospital. Gut Liver.

[10] Heneghan HM, Healy NA, Martin ST (2011). Modern management of pyogenic hepatic abscess: a case series and review of the literature. BMC Res Notes.

[11] Barnes PF, De Cock KM, Reynolds TN, Ralls PW (1987). A comparison of amebic and pyogenic abscess of the liver. Medicine.

